# Effect of nano-grain carbide formation on electrochemical behavior of 316L stainless steel

**DOI:** 10.1038/s41598-021-91958-x

**Published:** 2021-06-15

**Authors:** Chatdanai Boonruang, Wutipong Sanumang

**Affiliations:** 1 Department of Physics and Materials Science, Faculty of Science, Chiang Mai University, Chiang Mai, 50200 Thailand; 2 Center of Excellence in Materials Science and Technology, Chiang Mai University, Chiang Mai, 50200 Thailand

**Keywords:** Engineering, Materials science

## Abstract

The effect of low oxygen-partial pressured carburizing on relaxation process for 316L stainless steel is reported. Phase, morphology, and amount of compound formation during initial stage of carburizing are investigated using X-ray diffractometry (XRD) and X-ray photoelectron spectroscopy (XPS). The results show formation and development of surface multilayer with nano-grain-carbide (Cr_7_C_3_, Fe_7_C_3_, and/or Cr_3_C_2_) generation in the layer located below outermost protective layer. The relaxation process has been investigated using electrochemical impedance spectroscopy (EIS). Formation of nano-grain carbide(s) during carburizing causes deterioration effect on the electrochemical behavior of steel. However, the steel with large amount of carbide generation (carburized for 30 min) tends to have higher corrosion resistance (indicated by higher values of *R*_*cl*_ and *R*_*ct*_) than the smaller ones (10 and 20 min) due to the effect of phase, grain size, morphology, and amount of compound formation.

## Introduction

Metallic parts used in carbonaceous atmosphere (of carbon monoxide, methane, or other hydrocarbons) are subjected to carburizing. Carburizing is a long-standing corrosion problem in energy conversion and production systems of petrochemical industries including ethylene production^1–4^. Carburizing causes reaction between carbon from atmosphere and carbide forming elements in stainless steels such as Cr, Al, Si, and Fe. The carbon uptake and the formation of carbides promote local stresses that affect the mechanical properties of steels^1^. In operation, the components such as CO_2_-cooled nuclear reactor, furnace tube, and reformer or pyrolysis tubes^4–6^ are not only subjected to dry corrosion during in-service period but also wet corrosion in out-of-service one. Failure of the parts can lead to a leakage of chemicals which produces pollution problems and approximately 137 quadrillion joules of lost energy^2,7^. Austenitic stainless steels are extensively used in such carbonaceous atmosphere due to possession of higher carburizing resistance (resulted from lower diffusion coefficient of carbon) than ferritic stainless steels. Regarding the austenitic grades, corrosion and carburizing resistance of 316L stainless steel is superior to the 304 and 316 grades due to addition of Mo and possession of lower carbon content. Moreover, 316L stainless steel has nonmagnetism, good ductility, toughness, and weldability with lower price comparing to Ni-based alloys^4,8–10^. It is well known that the steels are susceptible to wet corrosion in marine environment due to the exposure to Cl^–^. In such wet or dry corrosion, the protective layer including Cr_2_O_3_ plays an important role in the corrosion protection of stainless steels^1,11–14^. Carburizing has a detrimental effect on the protective layer by a transport of carbon through Cr_2_O_3_ scale which therefore causes the corrosion problems^2,15,16^. Many research have been reported on only the effect of dry corrosion associated with carburizing, while the important effect of wet corrosion associated with electrochemical process is seemed to be ignored^1,2,15,16^. Our current study reports on effect of nano-grain carbide formation on electrochemical relaxation process for 316L austenitic stainless steel carburized in low oxygen-partial pressure. Such carbon rich environment corresponds to actual service environment that contains carbon monoxide, methane, or other hydrocarbons^1^. The effect of carburizing (phase, morphology, and amount of compound formation) on wet corrosion and critical period of service of the steel have been discussed.

## Methods

**Figure 1:**
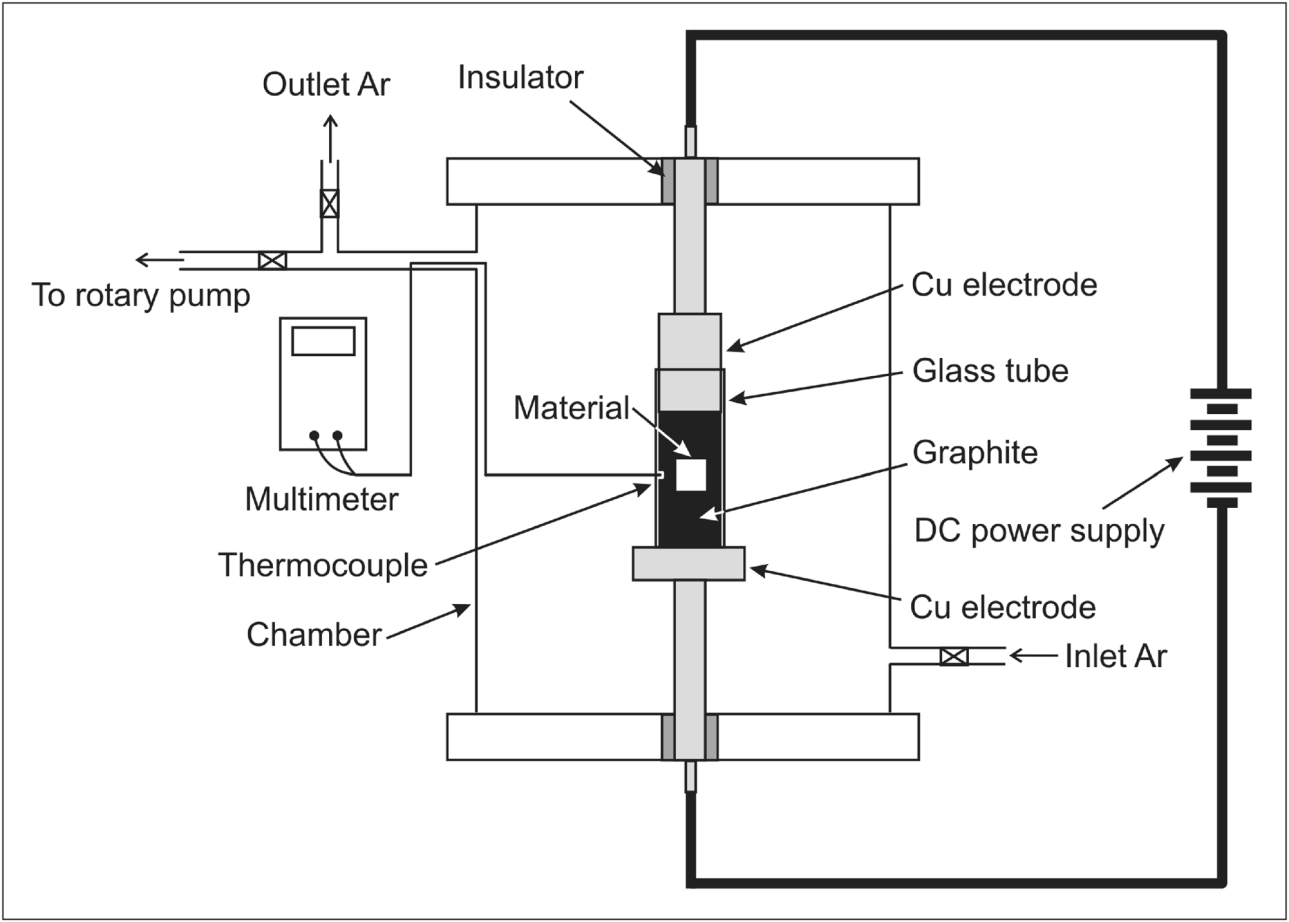
Configuration of current heating system used for carburizing.

Quarter-circle-shaped samples with 19 mm in diameter and 10 mm in height were prepared from (A276) annealed 316L stainless steel rod with chemical composition (wt%) of Fe–16.62Cr–12.00Ni–2.03Mo–0.012C–0.31Si–1.28Mn–0.037P–0.022S. The samples were ground and polished by 2000 grid SiC and 0.3 μm Al_2_O_3_. Carburizing was done using a current heating technique as the setup shown in Fig. 1. The sample was ultrasonically cleaned by methanol for 5 min and packed with a pressure of ~ 10.3 kPa in 20 μm-graphite powders placed between two copper electrodes in a glass tube. The chamber was evacuated in order to achieve a condition of low vacuum with absolute pressure of ~ 66 kPa and subsequently fed with 99.99%-purity Ar gas with a flow rate of 50 ml/min. DC current with a power of 300 W was applied to specimens for 10, 20, and 30 min for carburizing which resulted in carburizing temperatures of ~ 350, 550, and 600 °C, respectively. After treatment, the sample was removed and cooled down to ambient temperature in air.

Surface morphology of sample was analyzed by X-ray diffractometry (XRD) and X-ray photoelectron spectroscopy (XPS). XRD was performed using Mini Flex II (Rigaku) diffractometer with a Cu Kα X-ray source (1.5406 Å, 30 kV, and 15 mA) at 0.02° step size and 0.3 s step time. The area of 3 × 3 mm on surface of sample was sputtered by Ar^+^ with 1 keV ion energy for 20 s for removal of some contamination before characterized by XPS. The samples were examined using Kratos Axis ULTRA^DLD^ (Kratos) spectrometer equipped with a monochromatic Al Kα X-ray source (1486.6 eV). The base pressure in analysis chamber was approximately 5 × 10^–9^ torr. The X-ray source was used with the incidence angle of 45° to surface plane. The operation was done at 150 W (15 kV and 10 mA) with a spot size of 700 × 300 µm^2^ and initial photo energy of 1.4 keV. The binding energy of adventitious C 1 s peak at 285 eV was used for calibration of wavelength shift. The spectra were acquired (at a constant take-off angle of 90°) with the pass energy of 20 eV and analyzed with the energy step of 0.1 eV using VISION II (version 2.2.9) software. Impedance of samples were measured by electrochemical impedance spectroscopy (EIS). EIS measurement was performed using the Autolab-PGSTAT302N potentiostat (Metrohm Autolab B.V.) in aerated 3.5% NaCl solution at ~ 25 °C with a Ag/AgCl reference electrode and a platinum counter electrode incorporated with NOVA (1.11.0) software. The exposed surface area of working electrodes was ~ 0.9–1.0 cm^2^. Before EIS measurement, the open circuit potential (OCP) of working electrode was monitored for 2000 s or until a stable OCP was achieved. The amplitude of applied sinusoidal potential was 10 mV (r.m.s.) around the OCP and the frequency was controlled to be of between 100 kHz and 0.1 Hz. For each analysis condition, the EIS measurement was performed for at least three times by using a new sample in fresh solution. The closest single result to the average of multiple data was chosen to be the representative of each analysis condition. The electrochemical parameters of steels were determined by fitting the EIS experimental data using NOVA (1.11.0) software.

## Results and discussion

### Surface morphology analysis

**Figure 2:**
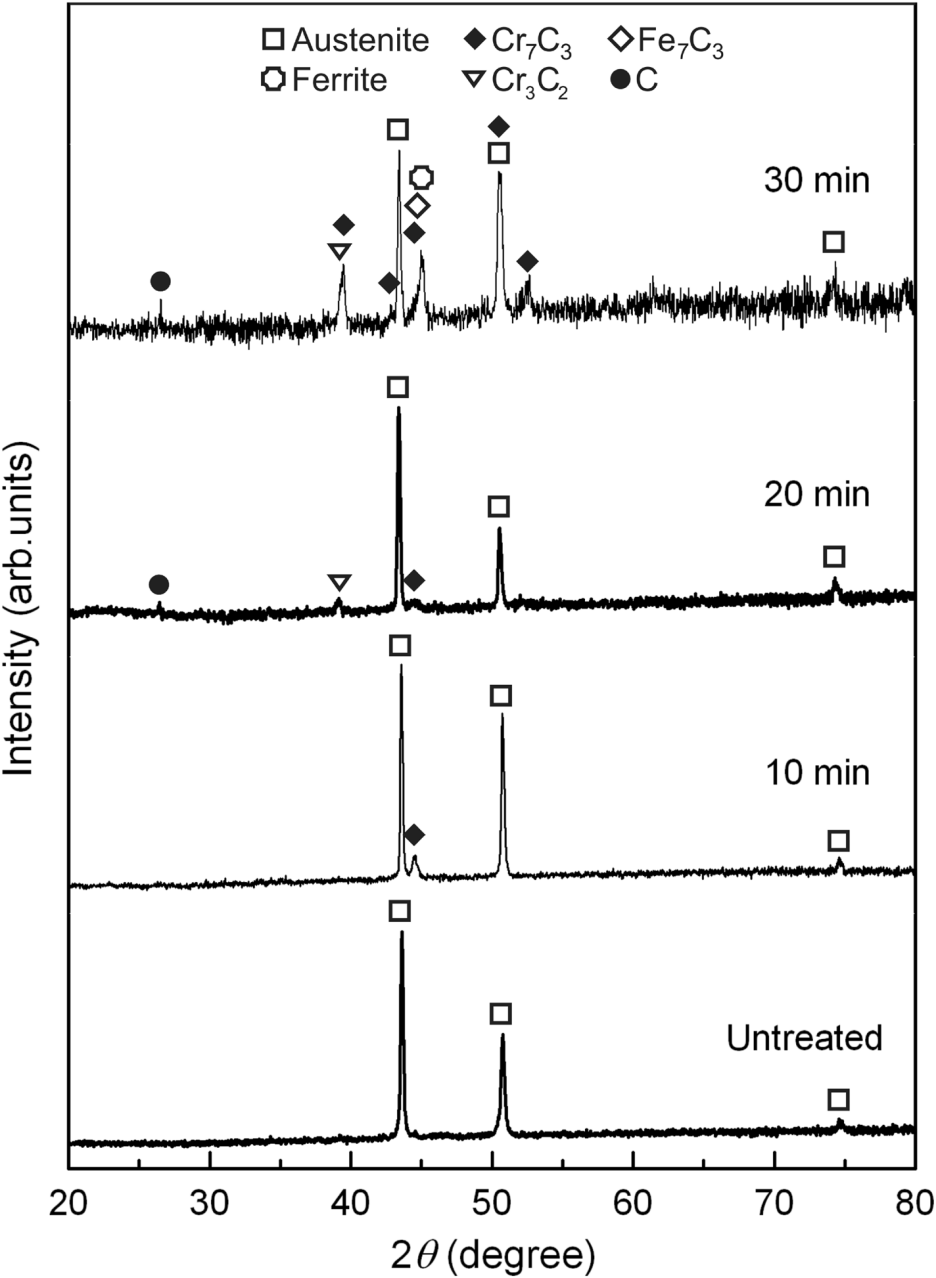
XRD patterns of 316L stainless steels treated at 300 W for 10 to 30 min and the untreated steel.

XRD patterns of the steels are shown in Fig. 2. For untreated steel, three peaks of austenite contributed by (111) (43.472°), (200) (50.673°), and (220) (74.677°) are observed. An additional primary peak of Cr_7_C_3_ contributed by (151) (44.599°) is present in the steel treated for 10 min. Peaks contributed by (121) (39.133°) and (003) (26.603°) of Cr_3_C_2_ and graphite, respectively, are observed in the steel treated for 20 min (Supplementary material 1). The peak intensity of Cr_7_C_3_ is lower than the steel treated for 10 min. The XRD pattern of steel treated for 30 min exhibits contribution of additional Cr_7_C_3_ peaks: (150) (39.491°), (112) (42.823°), (222) (50.673°), and (260) (52.551°). Primary peaks of ferrite (contributed by (110) (45.067°)) and Fe_7_C_3_ (contributed by (211) (44.855°)) are present close to the primary peak of Cr_7_C_3_ which results in peak convolution with asymmetric feature. For low strain material, grain size of the compound can be evaluated using Scherrer's equation:

1 $$D_{hkl} = K\lambda /\beta_{hkl} {\text{cos}}\theta$$

where *D*_*hkl*_ (Å), *K*, *λ* (Å), *β*_*hkl*_ (rad), and *θ* (rad) are average diameter of grain along the [*hkl*], Scherrer's constant (0.9 for assuming to be a spherical grain), wavelength of X-ray, full-width at a half-maximum of the (*hkl*) diffraction, and a half of diffraction angle, respectively^17–19^. The values of *β*_*hkl*_ and *θ* for calculation were obtained from the strongest non-overlapping peak of compound in the spectrum (Supplementary material 1). The sizes of nano-grains of Cr_7_C_3_ generated in steels treated for 10 (18.34 nm), 20 (10.45 nm), and 30 min (14.2 nm) were evaluated. The grain size of Cr_3_C_2_ generated in steel treated for 20 min was 19.75 nm. The thickness of compound layer generated in steels treated for 10 (~ 0.4 μm), 20 (~ 1.5 μm), and 30 min (~ 7.0 μm) were determined from cross-sectional images shown in Supplementary material 2.

**Figure 3:**
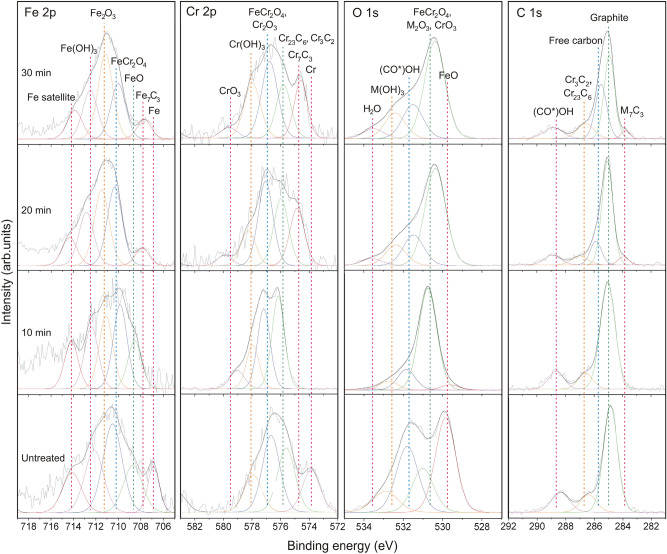
Fe 2p, Cr 2p, O 1s, and C 1s XPS spectra of 316L stainless steels treated at 300 W for 10 to 30 min and the untreated steel. The spectra (gray), convoluted peaks (black), and deconvoluted peaks (colors) are present. M in chemical formular of compound denotes Fe or Cr. The color of each deconvoluted peak corresponds to a color of dash line that indicates the position of binding energy of the chemical species.

XPS results in Fig. 3 show observation of carbides, oxides, hydroxides, carbon-oxide compound, graphite, and free carbon in the steels with binding energies corresponding to the compounds reported in previous research^1,12,20–33^. The peaks represent to Fe (~ 706.9 eV (Fe 2p))^12,20,24–27^ and Cr (~ 573.8 eV (Cr 2p))^12,20,22,23,25–27^ metals were only observed in untreated steel, while Fe_2_O_3_ peak (~ 711.2 eV (Fe 2p))^20,28^ was not observed. For the steel treated for 10 min, the peaks corresponding to FeO (~ 708.7 eV (Fe 2p) and ~ 529.8 eV (O 1s))^25,26,28^ were observed. For the samples treated for 20 and 30 min, the peaks associated with Cr_7_C_3_ (~ 574.7 eV (Cr 2p) and ~ 283.8 eV (C 1s))^1,22,33^, Cr_3_C_2_ (~ 575.8 eV (Cr 2p) and ~ 286.7 eV (C 1s))^23^, Fe_7_C_3_ (~ 707.8 eV (Fe 2p) and ~ 283.8 eV (C 1s))^1^, and free carbon (excess carbon which exists on the surface as graphite or amorphous carbon) (~ 285.7 eV (C 1s)^23,32^ were observed, while FeO peak was not observed. The peak contributed by CrO_3_ (~ 579.5 eV (Cr 2p))^26,27^ is present only in the treated steels. The peaks associated with Cr(OH)_3_ (~ 578.0 eV (Cr 2p), and ~ 532.6 eV (O 1s))^12,22,25–27^ were detected in both treated and untreated steels. The peaks corresponding to Fe(OH)_3_ (~ 712.5 eV (Fe 2p), and ~ 532.6 eV (O 1s))^27^ were detected in treated steels. The complete information about peak deconvolution are reported in Supplementary material 1.

The XRD and XPS results reveal surface morphology of steel in surface and near surface regions (by providing depth of analyses in micro- and nanoscales). For XPS results, it should be noted that Ni 2p signal was covered with background and is not present in this work. This reflects very low content of Ni in surface region and instability of Ni-containing compound in this carburizing condition. The XRD and XPS results show that the surface of untreated steel consists of a metallic bulk (solid solution of Cr in γ-Fe) which located under oxide-containing layers of Cr_2_O_3_, FeCr_2_O_4_, and FeO. Formation of FeO and FeCr_2_O_4_ generally occurs in a low chromium-content region. This region is produced by a slow diffusion of chromium at low temperature. The formation of FeCr_2_O_4_ is given by^15^

2 $${\text{Fe}} + {\text{Cr}}_{{2}} {\text{O}}_{{3}} + { 1}/{\text{2O}}_{{2}} = {\text{ FeCr}}_{{2}} {\text{O}}_{{4}}$$

Fe_2_O_3_ was not detected due to possession of lower thermodynamic stability than Cr_2_O_3_ at ambient temperature^34^. A presence of Cr_23_C_6_ reflects a precipitation of this phase which generally observed in austenitic stainless steel and aged 316L stainless steel^6^. The precipitation resulted from a reaction^35^

3 $${\text{23Cr }} + {\text{ 6C }} = {\text{ Cr}}_{{{23}}} {\text{C}}_{{6}}$$

The carbon-oxide compound (with binding energy corresponding to carboxyl ((CO*)OH)) observed in the spectra was due to surface contamination. Observation of hydroxides resulted from the exposure of steel to moisture as given by^36^

4 $${\text{M}}^{{{3} + }} + {\text{ 3H}}_{{2}} {\text{O }} = {\text{ M}}\left( {{\text{OH}}} \right)_{{3}} + {\text{ 3H}}^{ + }$$

where M denotes Fe or Cr. The Fe(OH)_3_ peaks are proposed to be not observed in the untreated steel due to Fe(OH)_3_ was less stable than Cr(OH)_3_ when the uncarburized steel exposed to air^37^. The peak observed at ~ 532.6 eV (O 1s) contributed by M(OH)_3_ is therefore proposed to be of Cr(OH)_3_ located at the outermost surface of untreated steel. The results are in agreement with previous research^38^. For treated steels, the M_2_O_3_ formed during carburizing at high temperature via a reaction^15^

5 $${\text{2M }} + { 3}/{\text{2O}}_{{2}} = {\text{ M}}_{{2}} {\text{O}}_{{3}}$$

when the steel was exposed to humid air after carburizing, the O–M bonds of M_2_O_3_ were weakened by H_2_O adsorption. The O atom of H_2_O molecule was bonded to M atom of M_2_O_3_, and another H–O bond was broken to form hydroxyl group (OH^−^)^39^. Formation of M(OH)_3_ is proposed to be given by

6 $${\text{M}}_{{2}} {\text{O}}_{{3}} + {\text{ 3H}}_{{2}} {\text{O }} = {\text{ 2M}}\left( {{\text{OH}}} \right)_{{3}}$$

For the steel treated for 10 min, no adding carbide produced by the carburizing process was observed at near surface by XPS. However, the nano-grain carbide was observed at a higher depth by XRD where the concentration of carbon in steel reached a critical level for Cr_7_C_3_ formation as given by^35^

7 $${\text{7Cr}}_{{{23}}} {\text{C}}_{{6}} + {\text{ 27C }} = {\text{ 23Cr}}_{{7}} {\text{C}}_{{3}}$$

This equation indicates higher stability of Cr_7_C_3_ (in comparison with Cr_23_C_6_) when the steel contains larger amount of carbon as reported by previous research^4,23^. Observation of additional Fe_2_O_3_ simultaneously with disappearance of Fe and Cr indicates oxidation of these metals which results in larger amount of oxide formation. The formation of Fe_2_O_3_ resulted from the reaction between outward-diffusing Fe^2+^ (through Cr_2_O_3_)^40^ and inward-diffusing O^2−^ at outermost surface of steel. The reduction of peak intensity in O 1s spectrum of low oxygen containing oxide such as FeO was due to possession of lower stability of this phase than the untreated steel. The reduction reflected larger amount of oxygen content in the surface of steel treated for 10 min. The high oxygen content also resulted in oxidation of Cr_2_O_3_ to form CrO_3_. The formation of carbides in steel treated for 20 min resulted from the reactions between C and compounds in steels^15,35,41^:

7 $${\text{7Cr}}_{{{23}}} {\text{C}}_{{6}} + {\text{ 27C }} = {\text{ 23Cr}}_{{7}} {\text{C}}_{{3}}$$

8 $${\text{7Cr}}_{{2}} {\text{O}}_{{3}} + {\text{ 6C }} = {\text{ 2Cr}}_{{7}} {\text{C}}_{{3}} + {\text{ 21O}}$$

9 $${\text{3Cr}}_{{2}} {\text{O}}_{{3}} + {\text{ 13C }} = {\text{ 2Cr}}_{{3}} {\text{C}}_{{2}} + {\text{ 9CO}}$$

10 $${\text{3Cr}}_{{7}} {\text{C}}_{{3}} + {\text{ 5C }} = {\text{ 7Cr}}_{{3}} {\text{C}}_{{2}}$$

The formation of Cr_3_C_2_ was difficult to be confirmed solely by XPS due to the binding energies of Cr 2p (~ 575.8 eV) and C 1s (~ 286.7 eV) for Cr_3_C_2_ are very close to other chromium carbide^23,41^. This carbide is proposed to be Cr_23_C_6_ which observed in uncarburized steel as described by Eq. (3). In thermodynamics theory of phase transformation, the phase existence and stability associate with formation (nucleation and growth) and decomposition of phase which can be indicated by amount of phase and grain size provided by XRD results. The XRD results show lower thermodynamic stability of Cr_7_C_3_ (indicated by the lower peak intensity and smaller grain size as described in Supplementary material 1) for the steel treated for 20 min when compared to 10 min due to some reasons. Cr_7_C_3_ in steel treated for 20 min decomposed and reacted with carbon to form Cr_3_C_2_ as given by reaction (10). The expense growth of Cr_3_C_2_ nanograins was able to suppress the Cr_7_C_3_ neighbors. The relative amount of Cr_3_C_2_ was larger than Cr_7_C_3_ after carburizing time of 20 min.The contents of Cr_7_C_3_ and Cr_3_C_2_ increased with increasing carburizing time. The results show that the highest carbon-containing carbide, Cr_3_C_2_, formed due to possession of high thermodynamics stability when chromium-containing material possessed large amount of carbon content at the suitable temperature for carbide formation. The large amount of carbon is reflected by a presence of a peak of graphite in which resulted from deposition of unreacted excess carbon. Disappearance of FeO and formation of Fe_7_C_3_ when the steels possessed high oxygen and carbon contents are proposed to be due to the reaction^1,42^:

11 $${\text{21FeO }} + {\text{ 9C }} + {\text{ 14Cr }} = {\text{ 3Fe}}_{{7}} {\text{C}}_{{3}} + {\text{ 7Cr}}_{{2}} {\text{O}}_{{3}}$$

Some nascent carbon (from carbon source) had not been involved in carbide formation and existed as free carbon. Some free carbon was amorphous and not seen in the XRD scan. Amount of free carbon on the steel surface tended to increase with increasing carburizing time as shown in the C 1s spectra. The steel treated for 30 min experienced the same compound formation as the 20 min with adding of ferrite. This ferrite was proposed to be the metal particles in oxide scale as reported in previous research^2^. These metal particles were produced by decomposition of FeCr_2_O_4_ as given by^2^

12 $$ {\text{FeCr}}_{{2}} {\text{O}}_{{4}} = {\text{a}}{-}{\text{Fe }} + {\text{ Cr}}_{{2}} {\text{O}}_{{3}} + { 1}/{\text{2O}}_{{2}} . $$

Carbon can diffuse through the α-Fe as an additional path. The formation of carbide was therefore attributed to a combined effect of traditional diffusion, grain boundary diffusion, and diffusion through the α-Fe particles. The XPS and XRD results therefore reveal surface morphologies of uncarburized steel and steels carburized under low oxygen-partial pressure as multilayer reported in Table 1. The multilayer of treated steels consisted of nano-grain carbide(s) generated in the compound layer that located between outermost protective layer and inner base stainless steel. The steel possessed development of phase and amount of carbide formation as carburizing time increased from 10 (Cr_7_C_3_) to 20 and 30 min (Fe_7_C_3_ + Cr_7_C_3_ + Cr_3_C_2_).

**Table 1:** Surface morphologies of uncarburized and carburized 316L stainless steels.

Steel	Surface morphology
Uncarburized	γ-Fe + Cr_23_C_6_ **|** FeO + FeCr_2_O_4_ **|** Cr_2_O_3_ + Cr(OH)_3_
10 min	γ-Fe + Cr_23_C_6_ **|** FeO + FeCr_2_O_4_ + Cr_7_C_3_ **|** Fe_2_O_3_ + Cr_2_O_3_ + CrO_3_ + Fe(OH)_3_ + Cr(OH)_3_
20 min	γ-Fe + Cr_23_C_6_ **|** FeCr_2_O_4_ + Fe_7_C_3_ + Cr_7_C_3_ + Cr_3_C_2_ **|** Fe_2_O_3_ + Cr_2_O_3_ + CrO_3_ + Fe(OH)_3_ + Cr(OH)_3_ + C
30 min	γ-Fe + Cr_23_C_6_ **|** FeCr_2_O_4_ + α-Fe + Fe_7_C_3_ + Cr_7_C_3_ + Cr_3_C_2_ **|** Fe_2_O_3_ + Cr_2_O_3_ + CrO_3_ + Fe(OH)_3_ + Cr(OH)_3_ + C

## Relaxation process and impedance

**Figure 4:**
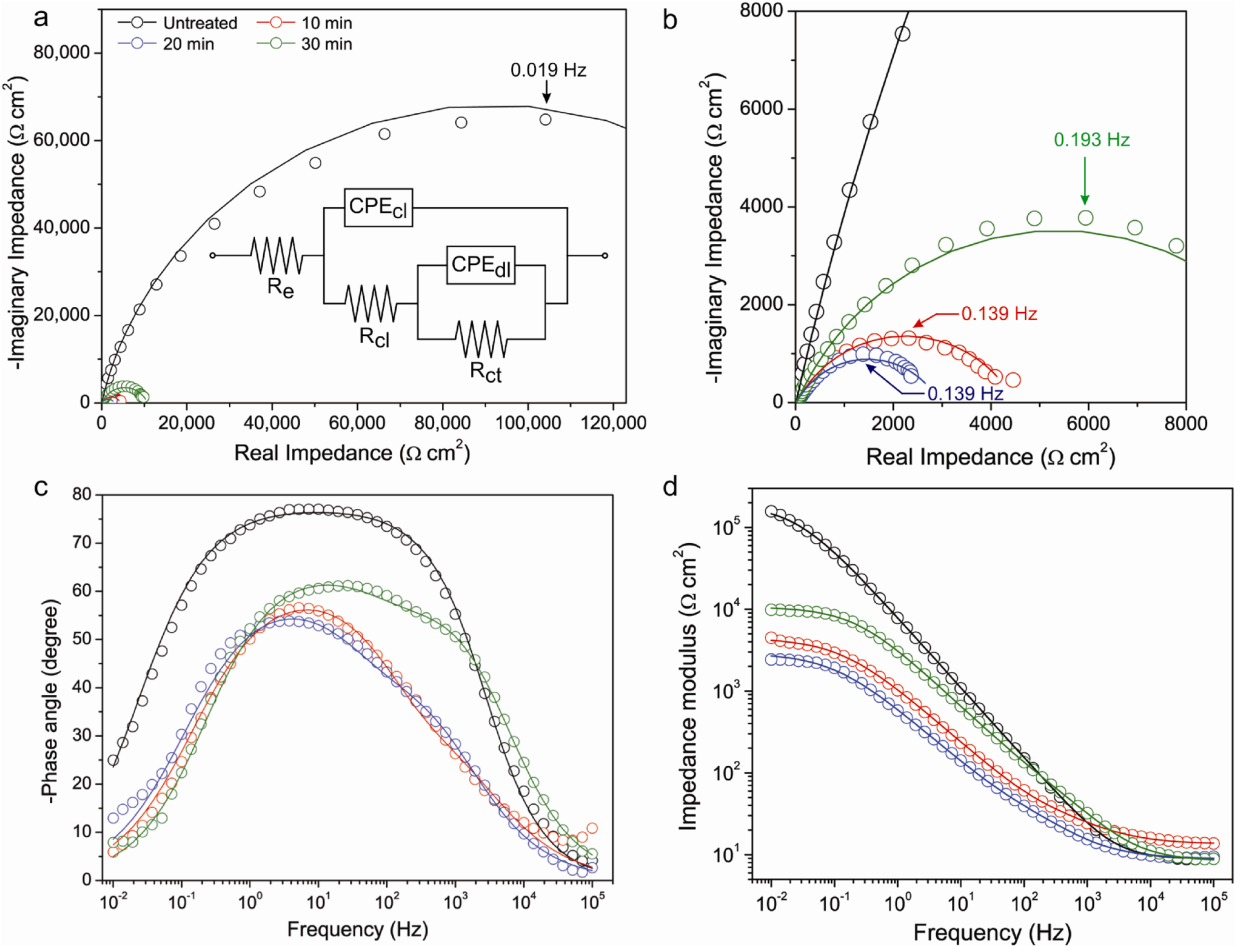
(**a**) Nyquist plots and the equivalent circuit used for simulation; (**b**) inset of Nyquist plots; c and d, Bode plots for 316L stainless steels carburized at 300 W for 10 to 30 min and the uncarburized steel. The EIS experimental data and simulated curves are represented by circles and lines, respectively.

Electrochemical behavior of the steels carburized at 300 W for 10 to 30 min and the uncarburized steel was investigated using EIS. The results (Fig. 4) and parameters (Table 2) obtained by curve fitting using the equivalent circuit with two time constants^43,44^ are shown. EIS parameters including constant phase element (CPE)-capacitive parameter (*Q*), capacitance associated with the CPEs (*C*_*CPEcl*_ and *C*_*CPEdl*_), passive (resistive) impedance of electrode (*Z*′), and *α* were calculated and reported. The value of *α* associates with microscopic surface roughness. The values of 0, 1, and –1, correspond to resistive, capacitive, and inductive behaviors, respectively^45,46^. *Z*′ is a summation of a polarization resistance of steel (*R*_*p*_) and Ohmic resistance (*R*_*e*_). *R*_*p*_ is obtained by summation of chemical layer resistance (*R*_*cl*_) and charge transfer resistance (*R*_*ct*_). *C*_*CPEcl*_ and *C*_*CPEdl*_ were calculated from *Q*_*CPEcl*_, *Q*_*CPEdl*_, *R*_*p*_, and *α* (using normal distribution) as given by^47^

13 $$C_{CPE} = Q^{{{1}/\alpha }} R_{p}^{{({1} - \alpha )/\alpha }}$$

**Table 2:** EIS parameters of the uncarburized and carburized steels obtained by curve fitting using the equivalent circuit and calculation.

Steel		Uncarburized	10 min	20 min	30 min
Parameters					
*OCP *(V)		− 0.123	− 0.455	− 0.392	− 0.351
*R*_*e*_ (Ω cm^2^)		9.83	13.4	8.90	8.32
CPE_dl_	*Q*_*CPEdl*_ (MΩ^–1^ cm^–2^ s^α^)	57.2	75.6	200	30.9
	*α*_*dl*_	0.720	0.748	0.690	0.702
*R*_*ct*_ (kΩ cm^2^)		50.6	4.44	2.86	10.5
CPE_cl_	*Q*_*CPEcl*_ (MΩ^–1^ cm^–2^ s^α^)	23.5	165	240	42.0
	*α*_*cl*_	0.858	0.670	0.699	0.766
*R*_*cl*_ (kΩ cm^2^)		154	0.0522	0.0684	0.232
*R*_*p*_ (kΩ cm^2^)		205	4.49	2.93	10.7
*Z′ *(kΩ cm^2^)		205	4.51	2.94	10.7
*C*_*CPEdl*_ (μF cm^–2^)		149	52.5	157	19.3
*C*_*CPEcl*_ (μF cm^–2^)		9.71	4.74	10.5	3.99
*χ*^2^		0.04769	0.07279	0.07747	0.02950
–*ϕ*_*max*_ (°)		76.4	56.1	54.3	61.3
*f*_*r*_ (Hz)		0.019	0.139	0.139	0.193

The relaxation process was clarified by Faradaic impedance of electrode which was simulated using the equivalent circuit with two time constants as shown in Fig. 4a. This circuit is appropriate for fitting of experimental impedance data of 316L stainless steel obtained in aerated media^44^. The impedance was constituted of real (*Z*′) and imaginary (*Z*′′) components corresponding to passive (resistive) and reactive (capacitive and/or inductive) contributions, respectively. The Faradaic impedance of steel was divided into two parts: chemical layer and double layer impedances which each contributed by *Z*′ and *Z*′′. These two impedances each consist of resistance (*R*) and capacitive impedance of a constant phase element which is given by^48^

14 $$Z_{CPE} = 1/Q(j\omega )^{\alpha }$$

where *Q*, *j*, and *ω* are CPE-capacitive parameter, $\sqrt { - 1}$, and angular frequency, respectively. The Nyquist plots of steels in Fig. 4a,b each show one time constant that indicates one relaxation or rate determining process for each steel. The relaxation frequencies (*f*_*r*_) of processes were in the range of below 1 Hz which indicated the rate determining relaxation process of oxygen adsorption on electrode surface^44,48^. *f*_*r*_ values for the carburized and uncarburized steels are in the order of magnitude o 0.1 and 0.01, respectively, as shown in Table 2. The adsorbed oxygen influenced on conversion and uncharged diffusion process. The conversion process associates with redox reaction that occurs in material-surface region. In redox reaction, metal atoms are oxidized and converted to metal ions and electrons. O atoms dissociated from O_2_ gas molecules (in electrolyte) adsorbing onto the surface are reduced by these electrons which results in generation of O^2−^^7,49^. In the testing environment of electrolyte with no carbon potential, Cr_3_C_2_ was less thermodynamically stable than Cr_7_C_3_ and underwent a larger amount of decomposition. The decomposition gave rise to the redox reaction or conversion process due to Cr^3+^ decomposed from the carbide was able to promote the reduction of adsorbing O atoms. The rising of redox reaction resulted in reduction of *R*_*ct*_ of the steel treated for 20 min when compared to 10 min. The uncharged diffusion process associates with O_2_ diffusion (perpendicular to the surface) in a stagnant gas layer that establishes an O_2_ concentration gradient (producing diffusion impedance) over the surface^7,50^. As active species, O_2_ diffused down the concentration gradient which arisen between the material surface and the location from material surface where the O_2_ concentration equal to a bulk solution. The bulk solution outside the stagnant layer possessed the higher O_2_ concentration than at the material surface. However, slow dissociation of O_2_ on the surface (which implied slow reduction rate) resulted in the accumulation of O_2_ and decrease in concentration gradient which gave rise to *R*_*ct*_ of the steel treated for 30 min. *R*_*cl*_ of the treated steels were lower than *R*_*ct*_ for two orders of magnitude which indicated small contribution of migration process in the corrosion of treated steels. The migration process associates with ion migration in chemical layer, and chemical layer impedance which depends on the structure of layer including phase, amount of compound, and grain size. The higher value of *R*_*cl*_ (~ 4.5 times) for the steel carburized for 30 min when compared to 10 and 20 min reflected the larger contribution of chemical layer impedance as reported in literature^8,51,52^. The results are in agreement with previous research^53^. XRD results show that Cr_7_C_3_ is the major compound formed in the carburized steels. The grain size of Cr_7_C_3_ therefore influenced on migration of metal ions which was largely contributed by a preferential diffusion pathway such as grain boundary. The small grain size in steel treated for 20 min contributed to the fast diffusion which corresponded to low *R*_*cl*_. However, *R*_*cl*_ of the steel treated for 20 min was higher than 10 min. It is proposed that the large thickness of compound layer and large grain size of Cr_3_C_2_ had some contribution in retardation of both traditional and grain-boundary diffusion in the steel treated for 20 min. The *R*_*cl*_ was therefore contributed by larger thickness of compound layer and grain-boundary diffusion associated with small Cr_7_C_3_ and large Cr_3_C_2_ grains. These contributions were larger than grain-boundary diffusion associated with large Cr_7_C_3_ grains in thin compound layer of the steel treated for 10 min. Even though the grain size of steel treated for 30 min was not the largest, the steel possessed the largest amount of Cr_7_C_3_ formation (as shown by XRD) and thickest compound layer (Supplementary material 2) which resulted in the highest *R*_*cl*_. A deviation from semi-circle shape of the Nyquist plots shown in Fig. 4b reflects a larger contribution of resistive than a capacitive one. The resistive contribution (corresponding to *Z′*) was therefore the main contribution in Faradaic impedance. Besides, a capacitance calculated from *Q*_*CPE*_ was able to imply the electrochemical susceptibility of steel. CPE-capacitance of chemical layer (*C*_*CPEcl*_) was attributed to the accumulation of charges (metal ions)^47^ in chemical layer of carburized steel. The steel with high *C*_*CPEcl*_ (such as carburized for 20 min) tended to be susceptible to corrosion. The CPE-capacitance of double layer (*C*_*CPEdl*_) of steels possessed different contribution in different relaxation processes. For the processes of oxygen adsorption and uncharged diffusion, high *C*_*CPEdl*_ indicated the large amounts of adsorption and accumulation of O_2_ gas on steel surface. The high *C*_*CPEdl*_ implied the slow dissociation of O_2_ or generation of O^2−^ which promoted the corrosion resistance of steel. For conversion process, high *C*_*CPEdl*_ indicated high conversion rate which promoted electrochemical susceptibility of steel. For the steel carburized for 20 min, large amounts of decomposition of Cr_3_C_2_ and reduction of adsorbing O atoms (promoted by small Cr_7_C_3_ grains) resulted in large amount of charge accumulation (of Cr^3+^ and O^2−^) in double layer which resulted in high value of *C*_*CPEdl*_. The contribution of *R*_*ct*_ in passive impedance (*R*_*p*_) of treated steels was larger than *R*_*cl*_ (for 2 orders of magnitude) as indicated by the values shown in Table 2. This reflects that oxygen adsorption had a greater effect than the migration process. The passive impedance reflecting corrosion resistance of steel therefore had an ascending order of the steel treated for 20 < 10 < 30 min. The order is in agreement with the results of potentiodynamic polarization reported in Supplementary material 3. The value of *R*_*p*_ for untreated steel was 1 to 2 orders of magnitude higher than the treated steels. The contribution of *R*_*cl*_ in *R*_*p*_ was larger than *R*_*ct*_ which reflected the large contribution of protective layer for untreated steel.

The results show that formation of nano-grain carbide(s) during carburizing had deterioration effect on the electrochemical behavior of steel due to disturbance of formation (as well as self-healing) of protective layer by delaying the growth of layer to achieve the critical thickness of protection^54,55^. However, the steel with large amount of carbide generation (treated for 30 min) tended to have higher corrosion resistance than the smaller ones (10 and 20 min) due to some reasons. Possession of smaller amount of ion migration as indicated by higher value of *R*_*cl*_, and larger amounts of adsorption and accumulation of O_2_ in a stagnant gas layer on surface of steel which resulted in reduction of concentration gradient and gave rise to *R*_*ct*_. The electrochemical behavior of carburized 316L austenitic stainless steel in this study exhibits the same trend as the 420 martensitic stainless steel of our previous work^7,54^ due to the formation of nano-grain-compound layer. The trend also shows some correspondence to previous research in which corrosion resistance of carburized 316L stainless steel (with formation of micro-grain-compound layer) was superior to the uncarburized steel. The enhancement of micro-grain-carburized layer resulted from retardation of mobility of oxygen-vacancy and metal-ion^52,55,56^. Besides, this also reflects the different electrochemical behavior between the carburized steels with nano- and micro-grain carbides. The corrosion resistance associated with electrochemical behavior of stainless steel is therefore mainly influenced by grain size, and amounts of hydroxide, oxide, and carbide formation of Cr. These compounds have an effect on determination of relaxation process of steel. For the sustainable operation of 316L stainless steel parts, the control of wet corrosion in period of formation of nano-grain carbide is highly recommended. The wet-corrosive environment and in-service period should be controlled and extended, respectively, until the nano-grain carbide has been developed to micro-grain carbide.

## Conclusions

Carburizing of 316L stainless steel in low oxygen-partial pressure resulted in formation of surface multilayer with nano-grain carbide(s). The carbide(s) generated in the compound layer that located between outermost protective layer and inner base stainless steel. The steel underwent development of phase and amount of carbide formation as carburizing time increased from 10 (Cr_7_C_3_) to 20 and 30 min (Fe_7_C_3_ + Cr_7_C_3_ + Cr_3_C_2_). Formation of nano-grain carbide(s) during carburizing had deterioration effect on the electrochemical behavior of steel due to disturbance of formation (as well as self-healing) of protective layer by delaying the growth of layer to achieve the critical thickness of protection. However, the steel with large amount of carbide generation (treated for 30 min) tended to have higher corrosion resistance than the smaller ones (10 and 20 min) due to some reasons. Possession of smaller amount of ion migration as indicated by higher value of *R*_*cl*_, and adsorption and accumulation of O_2_ in a stagnant gas layer on surface of steel which resulted in the reduction of O_2_-concentration gradient and gave rise to *R*_*ct*_. Phase, grain size, morphology, and amount of compound formation during carburizing had the effect on relaxation process of steel. For the sustainable operation of 316L stainless steel parts, the control of wet corrosion in period of formation of nano-grain carbide is highly recommended.

## Supplementary Information

Supplementary Information.
