# Supplementary material for: Effect of nano-grain carbide formation on electrochemical behavior of 316L stainless steel
Source: Sci Rep. 2021 Jun 15;11:12602. doi: 10.1038/s41598-021-91958-x (PMC8206347; doi:10.1038/s41598-021-91958-x)
Supplement: Supplementary file 1 — Supplementary Information. [file 41598_2021_91958_MOESM1_ESM.pdf]

Supplementary material

Effect of nano–grain carbide formation on electrochemical behavior of 316L stainless steel

Chatdanai Boonruang & Wutipong Sanumang

Supplementary material 1

Scherrer's equation is suitable to determine the grain size of carbide when the strongest non–overlapping peak of compound in the XRD pattern is present. For example, many peaks for the steel treated for 30 min were overlapped. We therefore choose a non–overlapping peak at 2θ = 52.551° for the calculation of grain size of Cr_7C_3. As shown in Fig. S.1, X'Pert HighScore (1.0a) software can enlarge scale of pattern and we can use the tools of "smooth" and coordinate reader to facilitate finding of the location and full–width at a half–maximum (FWHM) of the peak. The advantage of using Scherrer's equation is the peak diffracted by Cr_7C_3 grains located in wide area of sample leading to the calculated grain size would be close to the actual average grain size.

Formation of Cr_3C_2 (*Cmcm* orthorhombic structure[20]) cannot be confirmed solely by the XRD peak at 2θ = 39.133° contributed by (121) of Cr_3C_2. For the confirmation of formation of Cr_3C_2 in the steel treated for 20 min, we therefore use XRD combined with XPS and theory of carbide formation associated with an effect of carbon content. As we described formation of Cr_3C_2 using equation (10), it is reasonable to propose that the peak at 39.133° is a primary peak of Cr_3C_2 due to the intensity of primary peak of Cr_7C_3 at 44.599° was low. The contribution of Cr_7C_3 peak at 39.491° in convolution of the Cr_3C_2 peak would be therefore very low. Moreover, XPS results show the peaks of Cr_3C_2 (~575.8 eV (Cr 2p) and ~286.7 eV (C 1s)) in the spectra of steel treated for 20 min. Even though the peaks of Cr_3C_2

are very close to $Cr_{23}C_6$, the formation of Cr_3C_2 can be confirmed due to $Cr_{23}C_6$ is unstable when stainless steel contains high carbon.

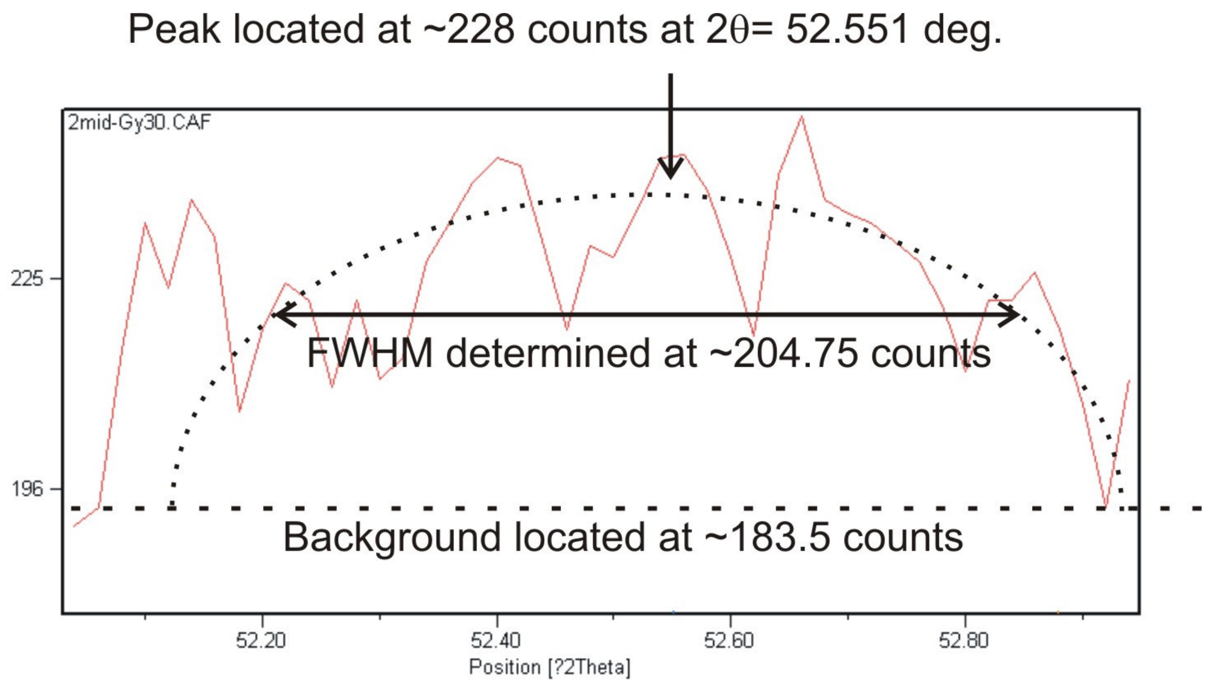

Figure S.1. Determination of the full–width at a half–maximum from the non–overlapping peak at 2θ = 52.551° in XRD pattern of 316L stainless steel treated at 300 W for 30 min.

Interpretation of the XRD results combined with the theory of nucleation and growth in phase transformation can explain thermodynamics stability of phase. In comparison between two conditions, i.e. 20 and 10 min, the different relative–peak intensity and FWHM of Cr_7C_3 reflected different amount and grain size of this phase. For 10 min, free energy of Cr_7C_3 formation reaction ($7Cr_{23}C_6 + 27C = 23Cr_7C_3$) was low, Cr_7C_3 was therefore stable and resulted in nucleation and growth. For 20 min, the increase in carbon content resulted in reduction of free energy of Cr_3C_2 formation reaction ($3Cr_7C_3 + 5C = 7Cr_3C_2$) or promotion of nucleation and growth (reflecting stability) of this phase. It is obvious that when this reaction proceeded, amount of Cr_3C_2 would increase, while Cr_7C_3 would relatively decrease due to formation (nucleation and growth) of Cr_3C_2 required decomposition of Cr_7C_3. It can be

therefore proposed that for stainless steel treated for 20 min, Cr_7C_3 possessed lower thermodynamic stability than Cr_3C_2. The stability of Cr_7C_3 in stainless steel treated for 20 min was lower than 10 min. The lower stability promoted the decomposition of Cr_7C_3. The amounts of Cr_7C_3 and Cr_3C_2 increased with increasing carburizing time. The relative amount of Cr_3C_2 was larger than Cr_7C_3 after carburizing time of 20 min. The relation between stability, peak intensity, and grain size is based on this theory of phase transformation.

Curve fitting of XPS spectrum can provide information about deconvoluted peaks associated with chemical species which present in sample. The data of deconvolution of steels are present in Table S.1. The data associate with the deconvoluted peaks in each spectrum.

Table S.1. The peak parameters of chemical species for different elements used in this study.

Element (photo electron core level)	Steel	Chemical species	Peak position (eV)	FWHM (eV)	%Area	Reference
Fe 2p	Uncarburized	Fe	706.86	1.20	10.4	20,24–27
		FeO	708.67	1.97	17.8	25,26
		$FeCr_2O_4$	710.49	1.97	33.3	25,26
		$Fe(OH)_3$	712.13	1.97	23.4	27
		Fe satellite	714.09	1.97	15.0	24,27
	10 min	FeO	708.52	1.42	15.3	25,26
		$FeCr_2O_4$	709.82	1.42	26.7	25,26
		Fe_2O_3	711.09	1.42	23.2	20,24,26–28
		$Fe(OH)_3$	712.38	1.42	19.8	27
		Fe satellite	714.16	1.42	15.0	24,27
	20 min	Fe_7C_3	707.87	1.42	6.4	1
		$FeCr_2O_4$	710.34	1.53	31.2	25,26
		Fe_2O_3	711.51	1.53	30.1	20,24,26–28
		$Fe(OH)_3$	712.87	1.53	21.2	27
		Fe satellite	714.37	1.53	11.1	24,27
	30 min	Fe_7C_3	707.68	1.42	7.7	1
		$FeCr_2O_4$	710.02	1.53	23.5	25,26
		Fe_2O_3	711.16	1.53	36.0	20,24,26–28
		$Fe(OH)_3$	712.52	1.53	20.7	27
		Fe satellite	713.88	1.53	12.1	24,27
Cr 2p	Uncarburized	Cr	573.85	1.42	19.2	12,20,22,23,25–27
		$Cr_{23}C_6$, Cr_3C_2	575.59	1.42	29.0	23
		Cr_2O_3, $FeCr_2O_4$	576.65	1.42	34.8	1,12,20–23,25–27
		$Cr(OH)_3$	577.93	1.42	16.9	12,22,25–27
	10 min	$Cr_{23}C_6$, Cr_3C_2	576.17	1.02	38.9	23
		Cr_2O_3, $FeCr_2O_4$	577.19	1.02	33.6	1,12,20–23,25–27
		$Cr(OH)_3$	577.96	1.02	18.4	12,22,25–27
		CrO_3	579.01	1.19	9.1	26,27

Element (photo electron core level)	Steel	Chemical species	Peak position (eV)	FWHM (eV)	%Area	Reference
	20 min	Cr_7C_3	574.83	1.19	22.5	1,22,33
		$Cr_{23}C_6$, Cr_3C_2	575.95	1.19	26.3	23
		Cr_2O_3, $FeCr_2O_4$	577.03	1.19	33.2	1,12,20–23,25–27
		$Cr(OH)_3$	578.24	1.19	13.8	12,22,25–27
		CrO_3	579.90	1.19	4.1	26,27
	30 min	Cr_7C_3	574.56	1.05	19.5	1,22,33
		$Cr_{23}C_6$, Cr_3C_2	575.77	1.19	20.4	23
		Cr_2O_3, $FeCr_2O_4$	576.78	1.30	32.3	1,12,20–23,25–27
		$Cr(OH)_3$	577.99	1.30	23.4	12,22,25–27
		CrO_3	579.63	1.30	4.5	26,27
O 1s	Uncarburized	FeO	529.86	1.19	39.7	25,26,28
		Cr_2O_3, $FeCr_2O_4$, CrO_3	530.99	1.26	19.4	22–28
		(CO*)OH	531.81	1.28	29.9	29–31
		$Fe(OH)_3$, $Cr(OH)_3$	532.93	1.48	10.9	12,22,25–27
	10 min	FeO	529.61	1.06	3.8	25,26,28
		Fe_2O_3, Cr_2O_3, $FeCr_2O_4$, CrO_3	530.74	1.06	73.4	22–28
		(CO*)OH	531.83	1.06	14.9	29–31
		$Fe(OH)_3$, $Cr(OH)_3$	532.72	1.06	6.5	12,22,25–27
		H_2O	533.79	1.06	1.3	23,24,26–28,30,31
	20 min	Fe_2O_3, Cr_2O_3, $FeCr_2O_4$, CrO_3	530.38	1.21	62.5	22–28
		(CO*)OH	531.48	1.21	19.7	29–31
		$Fe(OH)_3$, $Cr(OH)_3$	532.38	1.21	13.2	12,22,25–27
		H_2O	533.61	1.21	4.5	23,24,26–28,30,31
	30 min	Fe_2O_3, Cr_2O_3, $FeCr_2O_4$, CrO_3	530.40	1.21	58.1	22–28
		(CO*)OH	531.52	1.21	20.4	29–31
		$Fe(OH)_3$, $Cr(OH)_3$	532.42	1.21	15.4	12,22,25–27
		H_2O	533.53	1.21	6.0	23,24,26–28,30,31
C 1s	Uncarburized	Graphite	284.85	1.13	70.0	32
		$Cr_{23}C_6$, Cr_3C_2	286.39	1.31	13.4	23
		(CO*)OH	288.37	1.43	16.7	29–31
	10 min	Graphite	285.05	1.19	75.4	32
		$Cr_{23}C_6$, Cr_3C_2	286.61	1.19	11.4	23
		(CO*)OH	288.66	1.22	13.1	29–31
	20 min	Fe_7C_3, Cr_7C_3	283.94	0.88	4.8	1,22,33
		Graphite	285.06	0.88	63.2	32
		Free carbon	285.88	0.90	15.0	23,32
		$Cr_{23}C_6$, Cr_3C_2	287.02	1.31	8.0	23
		(CO*)OH	288.92	1.31	9.0	29–31
	30 min	Fe_7C_3, Cr_7C_3	283.82	0.67	3.4	1,22,33
		Graphite	284.91	0.85	43.4	32
		Free carbon	285.50	0.92	30.4	23,32
		$Cr_{23}C_6$, Cr_3C_2	286.61	1.35	12.4	23
		(CO*)OH	288.85	1.54	10.5	29–31

Supplementary material 2

Uncarburized and carburized 316L stainless steels were cross–sectioned, ground with 2000 grid SiC, polished with 0.3 μm alumina, and etched by a solution of H_2O (30 ml), HCl (20 ml), and HNO_3 (10 ml). SEM and EDS were carried out using JSM–6335F (Jeol) with 15 kV accelerating voltage, incorporated with INCA (version 4.06) software. The SEM images in Fig. S.2 exhibit formation of nano–grain–compound layer on surface of carburized steels. The thickness of nano–grain–compound layer for steels treated for 10 (~0.4 μm), 20 (~1.5 μm), and 30 min (~7.0 μm) were measured from the images. The layer thickness increased with increasing carburizing time which corresponded to amount of compound formation exhibited by XRD. EDS line scanning was done across the compound layer. However, small characteristic information of the compound layer was obtained due to low sensitivity (associated with the large probe size in comparison with the layer thickness) of this technique. As shown in Fig. S.3, the compound layer of steel carburized for 20 min composed of smaller amounts of Fe and Cr and larger amounts of C and O when compared to the steel substrate. The intensity of Ni spectrum was very low in both compound and substrate. It is proposed that the layer composed of some kinds of carbides and oxides of iron and chromium, and none of nickel compound. The line scanning for steels treated for 10 and 30 min exhibited the same trend as 20 min and have not been reported. The appropriate technique in characterization of morphology of compound layer was a combination use of XPS and XRD which were able to probe the layer from the depth of nano– to microscales. The interpretation of combination results is reported in Table 1.

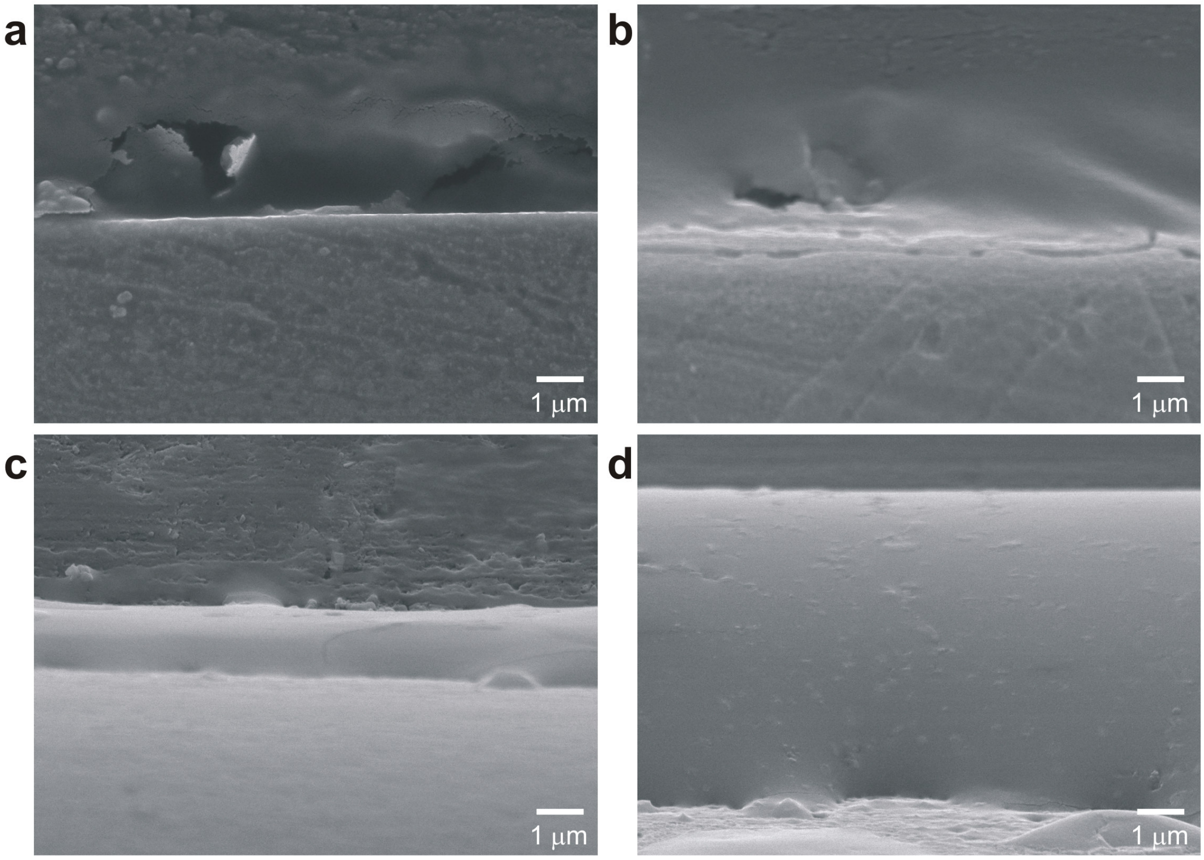

Figure S.2. Cross–sectional images of uncarburized 316L stainless steel (a) and the steels carburized at 300 W for 10 (b), 20 (c), and 30 min (d).

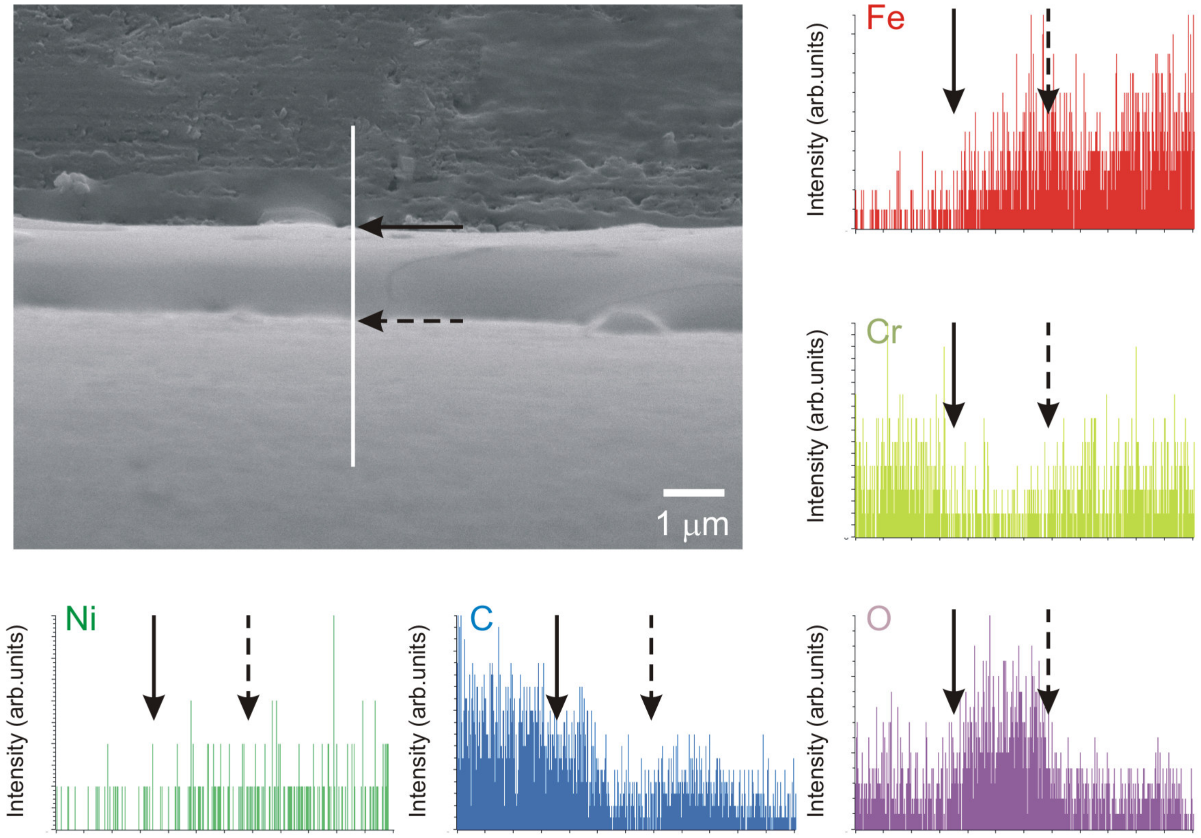

Figure S.3. EDS–line scanning was performed downward along the white line across a compound layer of steel carburized for 20 min. The positions of outermost surface (solid arrow) and substrate–compound interface (dash arrow) are indicated in each elemental spectrum.

Supplementary material 3

The corrosion kinetics of stainless steels was measured by potentiodynamic polarization (PP) using the same setup as EIS. PP was conducted at a scan rate of 10 mV/s over a scan range of –0.300 to +1.000 V with respect to OCP. The test duration for PP was 130 s. The polarization curve was analyzed using Tafel extrapolation method facilitated by NOVA (1.11.0) software. The results are shown in Fig. S.4 and Table S.2. The anodic polarization behavior of untreated steel consisted of active dissolution, unstable passivity, and transpassivity. The transpassivity was reflected by a rapid increase in the current density due to pitting which corresponded to previous research[57]. The behavior of treated steels was reflected by possession of active dissolution and stable passivity. The electrochemical kinetics of steels were indicated by E_{corr} and i_{corr} obtained by Tafel extrapolation. High value of i_{corr} indicates high rate of corrosion reaction. Low value of E_{corr} implies low corrosion resistance[52]. The untreated steel showed better corrosion behavior in comparison with the treated steels by exhibiting the lower i_{corr} and higher E_{corr}. Even though the i_{corr} values for treated steels were not much different, the difference was significant. The rate of corrosion reaction had an ascending order of the steel treated for 30 < 10 < 20 min. The order is corresponded to the descending order of Faradaic impedance of the steel treated for 30 > 10 > 20 min.

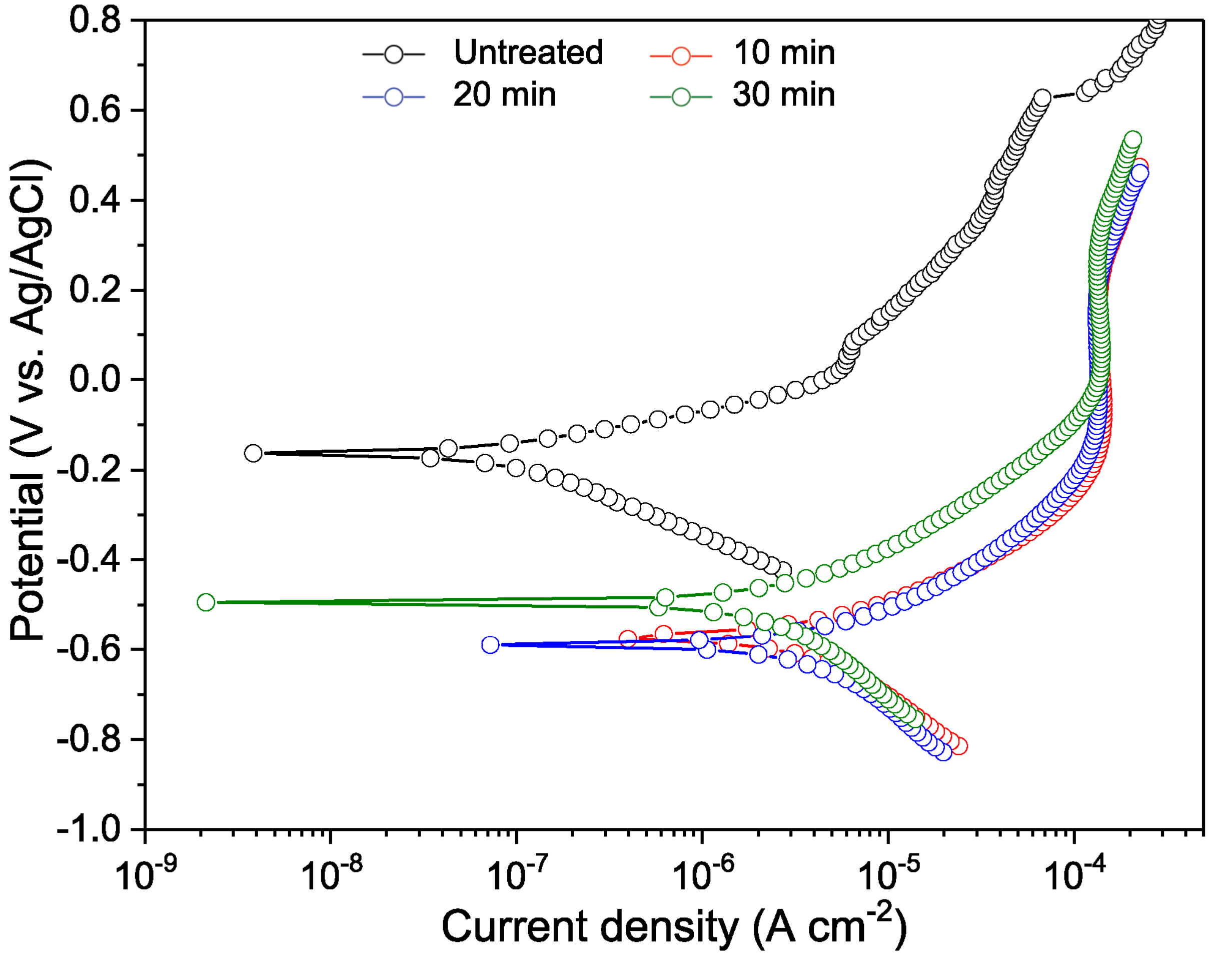

Figure S.4. Potentiodynamic polarization curves of 316L stainless steels treated at 300 W for 10 to 30 min and the untreated steel.

Steel / Parameters	Uncarburized	10 min	20 min	30 min
β_a	0.1813	0.4990	0.6185	0.6489
β_c	0.0930	0.2113	0.2464	0.2523
E_{corr} (V vs. Ag/AgCl)	–0.1700	–0.5737	–0.5778	–0.4908
i_{corr} (μA cm^{-2})	0.109	6.06	7.11	4.44
χ^2	3.34×10^{-14}	9.47×10^{-15}	3.23×10^{-14}	1.17×10^{-14}

Table S.2. The parameters for uncarburized and carburized steels obtained by Tafel extrapolation

References

[57] Yi, Y., Cho, P., Al Zaabi, A., Addad, Y. & Jang, C. Potentiodynamic polarization behavior of AISI type 316 stainless steel in NaCl solution. *Corros. Sci.* **74**, 92–97, https://dx.doi.org/10.1016/j.corsci.2013.04.028 (2013).
